# Effect of Medicaid Expansion on Visit Composition in a Louisiana Health Care System

**DOI:** 10.31486/toj.21.0106

**Published:** 2022

**Authors:** Diana Hamer, Deekshith Mandala, Glenn Jones, Gerald M. Knapp, Tonya Jagneaux

**Affiliations:** ^1^ Louisiana State University Health Sciences Center, School of Medicine, Baton Rouge Campus, Baton Rouge, LA; ^2^ Internal Medicine Residency Program, Our Lady of the Lake Regional Medical Center, Baton Rouge, LA; ^3^ College of Engineering, Louisiana State University A&M, Baton Rouge, LA

**Keywords:** *Emergency medical services*, *facilities and services utilization*, *healthcare disparities*, *Medicaid*, *Patient Protection and Affordable Care Act*, *primary health care*, *urgent care*

## Abstract

**Background:** In 2016, Louisiana expanded Medicaid to low-income adults under the Patient Protection and Affordable Care Act. By 2020, the uninsured rate of adults in Louisiana had dropped from 22.7% to 8.9%; however, few reports describe the effect Medicaid expansion has had on access and utilization of health care services in Louisiana.

**Methods:** For this study, we collected all-payer emergency department and clinic visits from one health care system in Louisiana from 2015 to 2019. We used a time series analysis to compare trends before and after Medicaid expansion in health insurance coverage and emergency department visit type.

**Results:** The changes in payer mix in the urgent care and primary care clinics and emergency departments after Medicaid expansion was driven by the uptake of Medicaid coverage in the previously uninsured. Medicaid expansion had a limited impact on the number of urgent care and emergent and nonemergent emergency department visits, but an increase in primary care visits was observed.

**Conclusion:** Medicaid expansion reduced uncompensated care in our patient population and expanded the access to primary care clinics. Ongoing research is needed to understand the effect of nonfinancial barriers to care on access to and utilization of services in Louisiana.

## INTRODUCTION

The Patient Protection and Affordable Care Act (ACA) was enacted in 2010. This comprehensive health care reform law expanded Medicaid to working individuals with incomes under 133% of the federal poverty level and created the federally facilitated Health Insurance Marketplace.^1^ The estimated effects of Medicaid expansion included an increase in Medicaid enrollment and Medicaid expenditures and a decrease in the rate of uninsured and uncompensated health care in states that expanded eligibility.^2,3^ In addition to coverage gains, studies examining telephone survey–based self-reported patient outcomes have shown an increase in access to care and health care services utilization,^4,5^ particularly for low-income adults,^6^ following Medicaid expansion. Additional survey studies found improvements in self-reported metrics including better overall health, better quality of care, and more outpatient visits in Medicaid expansion states.^5,7-9^ A systematic review found a large percentage of self-reported health assessments revealing gains in health and quality of care.^10^ Other research has found a discrepancy in self-reported data and administrative records with regard to post-Medicaid expansion health care service utilization.^11,12^ Thus, further exploration using nonsurvey research is needed.

A large body of literature exists on the effects of Medicaid expansion on emergency department (ED) utilization. Nationally, Medicaid recipients account for 40% of all ED visits and have the lowest percentage of ED visits resulting in hospital admission at 22.8%.^13^ Cheung et al found that Medicaid recipients report more barriers to care compared to privately insured individuals and demonstrated a positive correlation between the number of self-reported barriers and the number of ED visits.^14^ Administrative data revealed that Medicaid recipients have a higher probability of nonemergent and primary care (PC)–treatable ED visits compared to privately insured individuals.^15^ Studies also found newly enrolled Medicaid-recipient ED visits were primarily for nonemergent and outpatient care needs.^12,16^ Conversely, Medicaid expansion has not affected the volume of ED visits or ED admission rates but has principally impacted the payer composition.^17-20^ Post-Medicaid expansion, a larger percentage of ED visits was attributed to Medicaid recipients,^11,14,21^ accompanied by a decrease in the percentage of ED visits from uninsured patients.^21-24^ These studies do not examine the payer composition and patient visit volume of other health care services such as PC and urgent care (UC) clinics.

In 2016, Louisiana adopted the ACA Medicaid expansion to provide coverage for low-income adults.^25^ At the time of the expansion, Louisiana had the lowest Medicaid enrollment rate among eligible adults and the second highest number of newly eligible adults under ACA in the nation.^26^ Medicaid expansion recorded 468,414 newly enrolled from 2015 to 2020, and the uninsured rate dropped from 22.7% to 8.9%.^27,28^ In a 2019 report prepared by the Louisiana Department of Health, Diana et al found that ED utilization decreased by 20.7% between 2016 and 2018.^29^ Yet PC visits among the Medicaid expansion population remained low at 9.86% compared to 35.2% for the non-Medicaid population in 2018.^29^ The Diana et al report was limited to Medicaid beneficiaries who became eligible after the expansion.

For this analysis, we used time series models to quantitatively explore how Medicaid expansion impacted utilization across 3 health care settings: UC clinics, PC clinics, and the ED in a large health care system in Louisiana. We hypothesized that the insurance composition of the UC, PC, and ED visits and the nature of the ED visits were impacted by the Louisiana Medicaid expansion. In addition to the visit composition, we examined how the patient demographic composition changed over time in all 3 healthcare settings. We aimed to understand how the Louisiana Medicaid expansion affected health care utilization in a large Louisiana health care system.

## METHODS

### Study Design

This research design is a retrospective time series analysis structured to measure the impact of the 2016 Louisiana ACA Medicaid expansion on outpatient (UC or PC) and ED utilization. Adult patients at least 18 years of age who completed a UC, PC, or ED visit between June 1, 2015, and May 31, 2019, across hospitals and clinics affiliated with our health care system in the Greater Baton Rouge area were included in the analysis.

This study was approved with a waiver of informed consent by the Franciscan Missionaries of Our Lady University and the Louisiana State University Health Sciences Center institutional review boards.

### Source of Data

Since 2012, Our Lady of the Lake Regional Medical Center has been the main provider of indigent care in Baton Rouge, Louisiana. Within our health care systems, the EDs stabilize and treat all comers regardless of ability to pay or insurance status, as mandated under the Emergency Medical Treatment and Labor Act (EMTALA).^30^ The UC and PC clinics accept most major health insurance plans including Medicaid, and patients without insurance coverage are provided financial assistance. The sample consisted of visits to all UC and PC clinics affiliated with our health care system. The ED visits in the analysis consisted of 2 hospital-based EDs for the entire study period and a free-standing ED that opened to the public in November 2017. The study period was divided into 4 periods: the pre-Medicaid expansion period from June 1, 2015, to May 31, 2016; the first year post-Medicaid expansion from June 1, 2016, to May 31, 2017 (Year 1); the second year post-Medicaid expansion from June 1, 2017, to May 31, 2018 (Year 2); and the third year post-Medicaid Expansion from June 1, 2018, to May 31, 2019 (Year 3).

### Data Variables and Covariates

We used the electronic medical records, Epic (Epic Systems Corporation) and Cerner (Cerner Corporation), to obtain information on patients who completed a UC appointment, PC appointment, or ED visit. Data related to the encounter included date of clinic appointment or date of ED visit. *International Classification of Diseases* (ICD) 9/10 codes were abstracted for ED discharge diagnoses. Visits from patients without a Louisiana address were excluded from the analysis.

ED visits for which ICD-9 or ICD-10 codes were available were classified according to the NYU algorithm.^31^ This algorithm is based on the probability of each ICD-9 or ICD-10 code falling into 2 categories: emergent or nonemergent. Following Ballard et al,^32^ if the added probability of a nonemergent visit and PC-treatable visit was >50%, the visit was classified as nonemergent. If the sum of the preventable emergency probability and the nonpreventable probability was >50%, the visit was classified as emergent. Visits that had equal probability of being classified as nonemergent and emergent, those that were classified into the remaining categories (injury, drug, alcohol, or psych), and those that did not have an ICD-9/10 code were excluded from the analysis.^32^

### Data Analysis

Chi-square test of independence was used to compare the insurance status composition of the visits to the 3 health care settings and the ED visit classification across the 4 study periods, followed by post-hoc pairwise comparison with a Bonferroni-adjusted *P* value. We compared demographic composition in the year before and the years after Medicaid expansion using a chi-square Bonferroni-adjusted *P* value. A pairwise comparison was performed to identify proportions that significantly differed from each other at the 0.05 level. Statistically significant differences between study times are denoted by different superscripts in the tables. Statistical significance was set at *P*<0.05.

### Time Series Analysis

The number of UC, PC, and ED visits was aggregated by calendar month for each year. The unit of analysis was visits per month. We used a dummy variable denoting the 12 months before (pre-Medicaid expansion) and 36 months after (post-Medicaid expansion) Medicaid expansion to examine for the effect of Medicaid expansion. Linear interrupted time series analyses were used to examine for differences between pre-Medicaid and post-Medicaid expansion time periods for visits by insurance status. We used a first order autoregressive AR(1) model to correct for dependence in the data. Data were analyzed by insurance status. Similarly, an AR(1) model was used to examine the effect Medicaid expansion had on nonemergent and emergent visits to the ED.

## RESULTS

### Visits Over Time

#### Demographic Composition of Health Care Visits

A total of 164,431 UC, 816,254 PC, and 485,113 ED visits by patients who had a Louisiana address were abstracted and included in the analysis. Table 1 summarizes the total UC, PC, and ED visits pre-Medicaid expansion and during the 3 years thereafter. The insurance composition of UC visits (χ^2^ (9)=17191; *P*<0.001), PC visits (χ^2^ (9)=46366; *P*<0.001), and ED visits (χ^2^(9)=8170; *P*<0.001) significantly changed over time (Table 1). Private insurance enrollee visits to the UC clinics and ED significantly dropped across all 4 study periods, while PC visits significantly increased from pre-Medicaid expansion to Year 1 and Year 2. Medicaid visits in all 3 settings increased significantly across all 4 years, with the highest percentage gains occurring from pre-Medicaid expansion to Year 1 post-Medicaid expansion. Medicaid UC visits increased from 24.9% pre-Medicaid expansion to 45.9% in Year 1. In the same time period, PC visits increased from 9.6% to 15.8%, and ED visits increased from 18.9% to 34.6%. Conversely, self-pay visits decreased across all study periods, with the largest percentage decrease occurring from pre-Medicaid expansion (UC=49.5%, PC=12.8%, ED=28.6%) to Year 1 (UC=31.8%, PC=4.4%, ED=14.3%).

**Table 1.** Urgent Care, Primary Care, and Emergency Department Visits by Study Period and Insurance Status

		Post-Medicaid Expansion		
Visit Type/Insurance	Pre-Medicaid Expansion	Year 1	Year 2	Year 3
Urgent care^*^	43,231	40,870	40,404	39,926
Private	8,678 (20.1)^a^	5,428 (13.3)^b^	4,672 (11.6)^c^	5,331 (13.4)^b^
Medicare	2,398 (5.5)^a^	3,712 (9.1)^b^	4,860 (12.0)^c^	4,931 (12.4)^c^
Medicaid	10,776 (24.9)^a^	18,745 (45.9)^b^	21,998 (54.4)^c^	23,001 (57.6)^d^
Self-pay	21,379 (49.5)^a^	12,985 (31.8)^b^	8,874 (22.0)^c^	6,663 (16.7)^d^
Primary care^*^	169,603	195,923	223,576	227,152
Private	104,529 (61.6)^a^	108,573 (55.4)^b^	112,131 (50.2)^c^	110,115 (48.5)^d^
Medicare	27,003 (15.9)^a^	47,688 (24.3)^b^	72,356 (32.4)^c^	77,339 (34.0)^d^
Medicaid	16,287 (9.6)^a^	30,987 (15.8)^b^	32,734 (14.6)^c^	34,179 (15.0)^d^
Self-pay	21,784 (12.8)^a^	8,675 (4.4)^b^	6,355 (2.8)^c^	5,519 (2.4)^d^
Emergency department^*^	119,481	120,194	122,702	122,736
Private	31,394 (26.3)^a^	29,084 (24.2)^b^	27,690 (22.6)^c^	26,781(21.8)^d^
Medicare	31,358 (26.2)^a^	32,362 (26.9)^b^	32,771 (26.7)^a,b^	34,097 (27.8)^c^
Medicaid	22,564 (18.9)^a^	41,547 (34.6)^b^	49,679 (40.5)^c^	48,540 (39.5)^d^
Self-pay	34,165 (28.6)^a^	17,201(14.3)^b^	12,562 (10.2)^c^	13,318 (10.9)^d^

Note: Chi-square test of independence with Bonferroni correction between insurance types and study times; ^*^P<0.001. A different superscript letter denotes proportions that differ significantly from each other at the 0.05 level based on post-hoc pairwise comparison analysis for each study year pair: ^a^pre-Medicaid expansion vs Year 1, ^b^pre-Medicaid expansion vs Year 2, ^c^pre-Medicaid expansion vs Year 3, ^d^Year 1 vs Year 2, ^e^Year 1 vs Year 3, and ^f^Year 2 vs Year 3. Of note, no significant differences were identified between ^e^Year 1 vs Year 3 or ^f^Year 2 vs Year 3 for insurance types used for urgent care, primary care, or emergency department visits, so superscripts ^e^ and ^f^ do not appear in the table.

#### Emergency Department Visit Classification

Of the total number of ED visits in the study period, 192,193 (39.6%) were classified as nonemergent, 83,727 (17.2%) were classified as emergent, and the remaining 209,193 (43.1%) ED visits were classified into the other categories identified in the Methods section. ED visits classified as nonemergent significantly differed from emergent visits across all 4 study periods (χ^2^ (3)=147.53; *P*<0.001) (Table 2). Overall, the insurance composition of nonemergent visits (χ^2^ (9)=12085.9; *P*<0.001) and emergent visits (χ^2^ (9)=3753.6; *P*<0.001) changed. Emergent and nonemergent visits significantly decreased for private insurance visits pre-Medicaid expansion to Year 3. Medicare nonemergent visits were slightly less in the pre-Medicaid expansion period compared to Year 3, while emergent visits did not change over the study period. Nonemergent Medicaid visits increased from pre-Medicaid expansion to Year 2 followed by a slight drop in percentage composition in Year 3, and emergent visits increased in Years 1 and 2. Self-pay visits accounted for one-third of the nonemergent ED visits pre-Medicaid expansion and were reduced by half during the following 3 years. A significant reduction in self-pay emergent ED visits was also observed.

**Table 2.** Emergency Department Visit Classifications by Study Period and Insurance Status

		Post-Medicaid Expansion		
Emergency Department Visit Classification/Insurance	Pre-Medicaid Expansion	Year 1	Year 2	Year 3
Classification^*^	66,753	66,405	71,291	71,471
Nonemergent	47,428 (71.0)^a^	46,757 (70.4)^a^	48,925 (68.6)^b^	49,083 (68.7)^b^
Emergent	19,325 (29.0)^a^	19,648 (29.6)^a^	22,366 (31.4)^b^	22,388 (31.3)^b^
Nonemergent visits^*^				
Private	13,027 (27.5)^a^	11,857 (25.4)^b^	11,218 (22.9)^c^	10,909 (22.2)^c^
Medicare	9,414 (19.8)^a^	9,536 (20.4)^a^	9,904 (20.2)^a^	10,624 (21.6)^b^
Medicaid	9,638 (20.3)^a^	17,501 (37.4)^b^	21,739 (44.4)^c^	21,246 (43.3)^d^
Self-pay	15,349 (32.4)^a^	7,863 (16.8)^b^	6,064 (12.4)^c^	6,304 (12.8)^c^
Emergent visits^*^				
Private	5,205 (26.9)^a^	4,860 (24.7)^b^	5,130 (22.9)^c^	5,022 (22.4)^c^
Medicare	6,891 (35.7)^a^	6,861 (34.9)^a^	7,796 (34.9)^a^	7,986 (35.7)^a^
Medicaid	3,289 (17.0)^a^	6,024 (30.7)^b^	7,915 (35.4)^c^	7,669 (34.3)^c^
Self-pay	3,940 (20.4)^a^	1,903 (9.7)^b^	1,525 (6.8)^c^	1,711 (7.6)^d^

Notes: Chi-square test of independence with Bonferroni correction between insurance types and study times; ^*^P<0.001. A different superscript letter denotes proportions that differ significantly from each other at the 0.05 level based on post-hoc pairwise comparison analysis for each study year pair: ^a^pre-Medicaid expansion vs Year 1, ^b^pre-Medicaid expansion vs Year 2, ^c^pre-Medicaid expansion vs Year 3, ^d^Year 1 vs Year 2, ^e^Year 1 vs Year 3, and ^f^Year 2 vs Year 3. Of note, no significant differences were identified between ^e^Year 1 vs Year 3 or ^f^Year 2 vs Year 3 for insurance types for emergency department visits, so superscripts ^e^ and ^f^ do not appear in the table.

### Time Series Analysis

#### Urgent Care and Primary Care Setting

**Figure 1.**
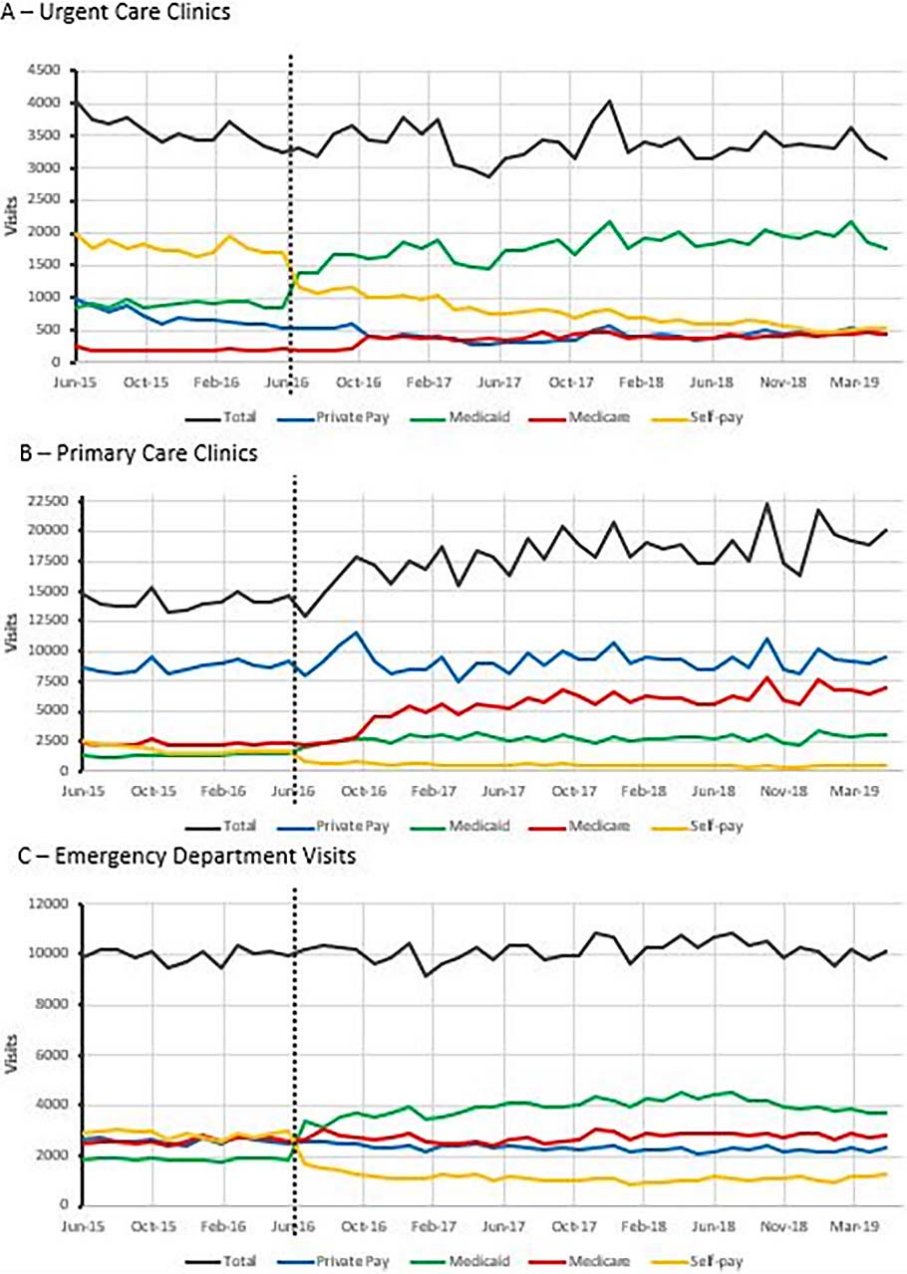
**Time series depiction of (A) total monthly urgent care clinic visits by insurance status, (B) total monthly primary care clinic visits by insurance status, and (C) total monthly emergency department visits by insurance status. Dashed vertical line indicates date of Medicaid expansion on June 1, 2016.** (For readers of the print publication, a color version of this figure is available at https://doi.org/10.31486/toj.21.0106.)

The time series analysis indicated no significant difference in total number of UC visits before Medicaid expansion compared to the 36 months after expansion (Table 3, Figure 1A). Private insurance UC visits were not significantly different between Medicaid expansion periods. Total Medicare and Medicaid visits significantly increased, while self-pay UC visits decreased post-Medicaid expansion. The total number of PC visits trended up over the study period, with more PC visits post-Medicaid expansion compared to the months prior (Table 3, Figure 1B). The monthly number of private insurance and Medicare visits did not change, while Medicaid increased and self-pay decreased.

**Table 3.** AR(1) Model Estimations by Insurance Status

Visit Type/Insurance	Pre-Medicaid Expansion, mean	Post-Medicaid Expansion, mean	Direction	Estimate^a^	Standard Error	*T* Test	*P* Value
Urgent care total visits	3,579.62	3,372.54	↓	−207.08	107.637	−1.924	0.061
Private	596.58	581.30	↓	−15.08	72.95	−0.21	0.835
Medicare	319.47	340.10	↑	20.63	44.38	44.38	<0.001
Medicaid	944.97	1,773.72	↑	828.74	91.91	9.01	<0.001
Self-pay	1,464.47	937.27	↓	−527.13	91.69	−5.74	<0.001
Primary care total visits	14,269.80	18,037.19	↑	3,767.38	632.56	5.96	0.001
Private	8,742.92	9,192.77	↑	449.85	257.88	1.74	0.088
Medicare	4,624.67	4,653.33	↑	28.66	725.72	0.04	0.969
Medicaid	1,387.00	2,755.01	↑	1,386.02	88.47	15.66	<0.001
Self-pay	1,764.79	942.78	↓	−822.01	113.16	−7.26	<0.001
Emergency department total visits	9,952.91	10,152.81	↑	199.90	135.84	1.47	0.148
Private	2,600.80	2,318.66	↓	−282.13	45.66	−6.179	<0.001
Medicare	2,608.48	2,762.164	↑	153.68	66.51	30.65	<0.001
Medicaid	2,145.07	3,792.24	↑	1,647.16	174.15	9.458	<0.001
Self-pay	2,744.72	1,199.68	↓	−1545.4	96.01	−16.09	<0.001
Nonemergent emergency department total visits	3,940.66	4,027.85	↑	87.19	87.64	0.995	0.325
Private	1,077.28	943.16	↓	−134.12	21.80	−6.151	<0.001
Medicare	786.39	836.52	↑	50.13	33.58	1.49	0.143
Medicaid	942.65	1,633.33	↑	690.68	86.88	7.95	<0.001
Self-pay	1,252.85	552.89	↓	−699.98	36.54	−19.15	<0.001
Emergent emergency department total visits	1,646.02	1,785.76	↑	139.74	75.83	1.84	0.072
Private	435.85	416.27	↓	−19.58	12.12	−1.61	0.113
Medicare	574.57	632.0	↑	57.43	28.0	2.05	0.046
Medicaid	316.63	582.72	↑	266.09	43.73	6.08	<0.001
Self-pay	331.24	137.46	↓	−193.78	10.08	−19.23	<0.001

^a^Difference between the mean for pre-Medicaid expansion months and post-Medicaid expansion months. Negative estimate indicates a decrease in visits between the 2 time periods while a positive estimate indicates an increase.

#### Emergency Department Setting

No significant change occurred in the total monthly number of ED visits prior to and after Medicaid expansion. However, significant changes were seen in the private, Medicare, Medicaid, and self-pay ED visits (Table 3, Figure 1C). The number of monthly private pay visits decreased during the time period. Medicaid increased sharply from pre- to post-Medicaid expansion, while self-pay decreased sharply.

**Figure 2.**
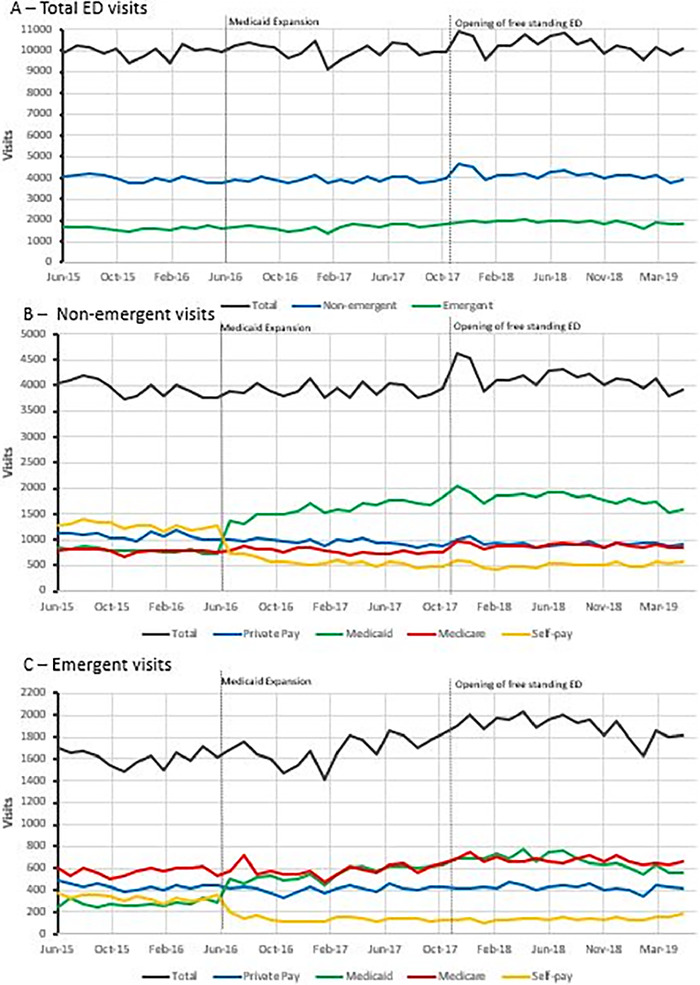
**Time series depiction of (A) emergency department (ED) monthly visits by ED visit classification, (B) nonemergent ED visits by insurance status, and (C) emergent ED visits by insurance status. Dashed vertical lines indicate date of Medicaid expansion on June 1, 2016, and opening of a free-standing ED on November 15, 2017.** (For readers of the print publication, a color version of this figure is available at https://doi.org/10.31486/toj.21.0106.)

The time series analysis did not reveal any changes in the total monthly emergent and nonemergent ED visits from pre-Medicaid expansion to the 36 months after expansion (Figure 2A). However, when visits were analyzed by payer group, the time series analysis revealed that monthly Medicaid visits increased, while monthly self-pay visits decreased by similar estimates after Medicaid expansion for both nonemergent and emergent ED visits. (Table 3, Figures 2B and 2C). Private insurance nonemergent monthly visits decreased after Medicaid expansion, while Medicare emergent monthly visits increased slightly.

## DISCUSSION

This study examined the effect of Medicaid expansion on visits and patient composition in the UC and PC clinics and the ED. We report several important findings. First, the insurance composition of UC, PC, and ED visits changed significantly over the 3 years post-Medicaid expansion. Second, Medicaid expansion did not significantly impact the number of UC and ED visits; however, PC visits increased. Third, no change occurred in the total number of nonemergent or emergent ED visits post-Medicaid expansion compared to the year prior.

The impact Medicaid expansion has had on health care visits attributed to Medicaid recipients and the uninsured in our study population is consistent with reports examining the effects of Medicaid expansion in Louisiana and other expansion states.^2,18,28,29^ The number of Medicaid-covered UC, PC, and ED visits increased from 24.9% to 45.9%, 9.6% to 15.8%, and 18.9% to 34.6%, respectively, the first year after Medicaid expansion, with lesser gains observed in subsequent years. Conversely, self-pay visits decreased in all 3 settings in the first year post-Medicaid expansion and continued to decrease in subsequent years. The reduction in self-pay visits highlights the main goal of the ACA of decreasing uninsured rates and uncompensated medical care.^33^ Opponents of the ACA expected that Medicaid expansion would discourage and exclude insured individuals, particularly Medicare recipients, from an already strained health care system, particularly in the PC setting.^34-37^ However, in the years after Medicaid expansion, we found a significant increase in Medicare-attributed UC and ED visits. Such increases are likely attributable to expanded preventive benefits and improvements in Medicare Part D spending, benefits available under Medicare Advantage plans, and limits on out-of-pocket costs for Medicare Advantage enrollees provisioned under the ACA.^38^ In contrast, the percentage of private insurance visits decreased following Medicaid expansion in the UC and ED, while PC visits increased. Private insurance continues to be the predominant form of insurance in the United States.^39^ Studies have demonstrated that healthy adults were more likely to gain private coverage, while nonhealthy adults enrolled in Medicaid in expansion states.^40^ Furthermore, type of coverage may have shifted from private insurance to Medicaid. However, this hypothesis is not supported by research that demonstrates stable per capita hospitalization for privately insured patients.^41^ Little evidence is available on the effects that Medicaid expansion has had on private insurance visits to the UC and PC clinics and the ED. Our study suggests that further research is needed to account for this trending decline in our study population.

We found that the 2016 Louisiana Medicaid expansion did not significantly impact the monthly total ED visits, including emergent and nonemergent visits, or UC visits but did increase PC visits during the 36 months post-Medicaid expansion. Large changes in payer mix were observed over time in the UC, PC, and ED settings post-Medicaid expansion. The ED trends found here agree with stable ED visit volume and ED admission rates found in administrative and survey data for other Medicaid expansion states.^17-20^ Using health care system data, we found that after Medicaid expansion, Medicaid visits accounted for approximately 40% of the nonemergent visits to the ED but only 15% of the visits to PC clinics. Medicaid recipients likely still rely heavily on the ED for outpatient needs. Although Medicaid expansion decreases financial barriers to care, other barriers remain, such as access to clinics, transportation, PC clinic hours, and timely appointment scheduling.^14,29,42^ These barriers could potentially explain the high percentage of UC visits attributed to Medicaid in the years post-Medicaid expansion.

### Limitations

This study has several limitations. First, we examined visits to the ED and outpatient clinics of one health care system in one region of south Louisiana. Therefore, whether these findings can be expanded to other populations within and outside of Louisiana is unclear. However, these data are consistent with findings from Medicare administrative and survey-level data among other ACA states, suggesting the results do generalize. Second, the retrospective nature of this study, which is based on administrative data, prevents us from comparing our findings to survey-based utilization behavior data before and after Medicaid expansion. Third, the analysis did not include potential confounders such as population changes or other external factors that could have affected health care–related visits. Finally, we did not explore nonfinancial determinants of health within our population, such as transportation access, level of education, and patient proximity to health care access points. These factors impact health care access and utilization and necessitate further investigation to fully understand the effects of Medicaid expansion in a southern state.

## CONCLUSION

Our findings indicate that changes in payer mix after Medicaid expansion in all 3 health care settings were mostly driven by self-pay patients gaining Medicaid coverage. These gains in Medicaid coverage were principally observed the first year post-Medicaid expansion in Louisiana. The reduction in self-pay health care visits and patients highlights the impact Medicaid expansion had on uncompensated care. Furthermore, visits to the ED and UC clinics did not significantly increase after Medicaid expansion, but the increase in PC visits and patients suggests expanded access to PC services. More research is needed to explain the patient demographic differences seen in UC and PC clinics and ED utilization and to measure the effect of nonfinancial barriers to health care to determine the full impact of the 2016 Louisiana Medicaid expansion.

## Notes

*This article meets the Accreditation Council for Graduate Medical Education and the American Board of Medical Specialties Maintenance of Certification competencies for Patient Care, and Systems-Based Practice.*
